# Developmental venous anomalies on fetal magnetic resonance imaging: prevalence and reproducible radiological phenotypes

**DOI:** 10.1007/s00247-026-06704-0

**Published:** 2026-07-13

**Authors:** Stephanie Libzon, Moran Hausman-Kedem, Nira Schneebaum-Sender, Li-Tal Pratt, Zvi Leibovitz, Gustavo Malinger, Karina Krajden Haratz, Shelly I. Shiran, Catherine Garel, Liat Ben Sira

**Affiliations:** 1 Pediatric Neurology Institute, Dana-Dwek Children’s Hospital, Tel Aviv Sourasky University Medical Center (Ichilov), 6 Weizmann Street, Tel Aviv, 6423906 Israel; 2 Gray Faculty of Medical and Health Science, Tel Aviv University, Tel Aviv, Israel; 3 Pediatric Radiology, Department of Radiology, Dana-Dwek Children’s Hospital, Tel Aviv Sourasky University Medical Center (Ichilov), Tel Aviv, Israel; 4 Obstetrics & Gynecology Ultrasound Unit, Bnai Zion Medical Center, Haifa, Israel; 5 Division of Ultrasound in Obstetrics and Gynecology, Lis Maternity and Women’s Hospital, Tel Aviv Sourasky University Medical Center (Ichilov), Tel Aviv, Israel

**Keywords:** Developmental venous anomalies, Hemorrhage, Fetal, Magnetic resonance imaging, Prenatal diagnosis, Vascular malformation

## Abstract

**Background:**

Developmental venous anomalies are the most common cerebral vascular malformations but remain underrecognized in fetuses. Accurate prenatal identification is important for diagnosis and counseling.

**Objective:**

This study aimed to describe the prevalence of developmental venous anomalies on fetal MRI and characterize their distinctive MRI patterns.

**Materials and methods:**

A retrospective study of all fetal MRIs performed between January 2017 and June 2025. Cases with documented or suspected developmental venous anomalies were extracted, including a systematic re-evaluation of studies with susceptibility artifacts, to identify developmental venous anomalies not categorized initially as such. Two independent reviewers assessed the number, location, and drainage pattern (superficial, deep, or mixed) of developmental venous anomalies. Associated abnormalities, including varix thrombosis, parenchymal hemorrhage and volume loss, and vascular malformation, were recorded. Developmental venous anomalies with associated abnormalities or multiple developmental venous anomalies were classified as complicated developmental venous anomalies.

**Results:**

Among 3,932 fetuses, 18 (0.46%) had developmental venous anomalies (median gestational age 33 weeks (IQR, 32, 33); 13 males). Developmental venous anomalies were frontal in 10 (55.6%), parieto-occipital in 7 (38.9%), and parietal in 1 (5.6%); 14 (77.8%) were right-sided. Deep drainage occurred in 8/18 (44.4%), superficial in 4/18 (22.2%), and mixed in 6/18 (33.3%). Two reproducible MRI patterns were identified: the “fisherman’s-rope” sign in superficial frontal developmental venous anomalies, and ventricular-wall T2 hypointensity in developmental venous anomalies with deep drainage. Eight fetuses (44.4%) had a complicated developmental venous anomaly. Multiple developmental venous anomalies (3/18, 16.7%) were associated with varix thrombosis (2/3 vs 0/15, *P*=0.02).

**Conclusion:**

Fetal developmental venous anomalies demonstrate two reproducible MRI patterns that enable confident diagnosis and help in differentiating from hemorrhage and other vascular malformations.

**Graphical abstract:**

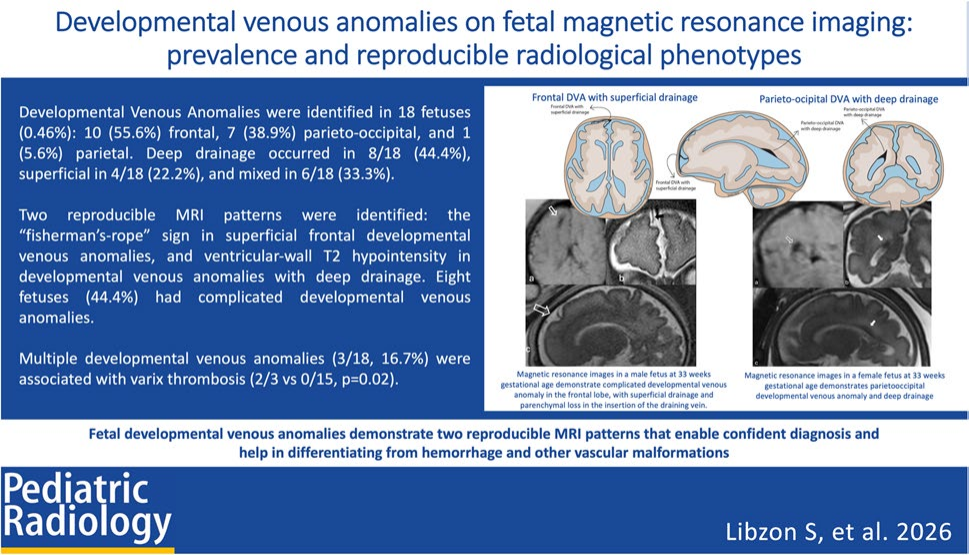

**Supplementary Information:**

The online version contains supplementary material available at 10.1007/s00247-026-06704-0.

## Introduction

Developmental venous anomalies are the most common cerebral vascular malformation, traditionally regarded as benign incidental findings on neuroimaging [1, 2]. They are characterized by radially oriented medullary veins converging into a central collecting vein, creating the classic “caput-medusae” appearance best visualized on contrast-enhanced T1-weighted or susceptibility-weighted imaging (SWI) sequences [1, 3, 4].

Although common, the recognition of developmental venous anomalies during the fetal period remains limited. Recent advances in high-resolution fetal MRI have enabled their identification in utero, where they typically appear on SWI, with an occasionally visible collecting vein or subtle parenchymal changes [1, 5, 6]. Such findings support the concept that developmental venous anomalies are congenital in origin, likely forming due to developmental arrest of veins during the late first trimester, representing a compensatory drainage pattern [2]. It was proposed that developmental venous anomalies develop postnatally as adaptive responses to localized changes in architecture and drainage patterns [7]. In contrast, fetal imaging studies have directly visualized developmental venous anomalies before birth, reinforcing the hypothesis of an in-utero origin [1, 5]. The ability to identify developmental venous anomalies prenatally raises important questions regarding their development, clinical implications, and associations with structural brain abnormalities. Most neonatal developmental venous anomalies are discovered incidentally on imaging performed for other reasons. In one study, developmental venous anomalies were detected in about 1.9% of neonatal brain scans [1].

Developmental venous anomalies are mostly asymptomatic, with an overall good prognosis and a benign course when isolated. They may be seen in conjunction with other focal findings, such as cavernous malformations [2, 8]. However, unlike the stable appearance in adults, some studies suggest that neonatal developmental venous anomalies may undergo dynamic changes in their anatomical extent or flow velocity over time [1]. This may partly explain why they are rarely diagnosed in this age group and are more often discovered incidentally later in life.

In a neonatal series [1], approximately one-third of cases, neonatal developmental venous anomalies coexist with parenchymal abnormalities within their drainage territory, including restricted-diffusion, venous congestion, and cortical malformations like polymicrogyria [1]. These “complicated developmental venous anomalies,” typically associated with multiple or large collector veins, may reflect increased hemodynamic burden or impaired venous drainage. Although uncommon, such lesions highlight that developmental venous anomalies can occasionally contribute to local brain injury [1].

Despite neonatal data, the prenatal imaging spectrum of developmental venous anomalies remains poorly defined [5, 6]. The ability of fetal MRI to predict associated parenchymal changes remains unclear. A more comprehensive characterization of fetal MRI features, including location, drainage pattern, and parenchymal associations, could refine prenatal diagnosis and counseling. We aim to describe the prevalence of developmental venous anomalies in a large cohort of fetuses that performed prenatal MRI, and to identify reproducible imaging patterns that enhance diagnostic confidence, with an attempt to distinguish accurate differentiation from hemorrhage, even without the use of the “gold standard,” including SWI and enhancement, thus improving prenatal counseling and future MRI protocol development.

## Methods

### Study design and population

This retrospective study was approved by the institutional review board (0131-24), with informed consent waived for this anonymized retrospective analysis. The fetal MRI database at Tel-Aviv University Medical Center was retrospectively reviewed (January 2017-June 2025). Since 2017, each scan routinely included at least one hemorrhage-sensitive sequences - gradient echo (GRE)-based T2-weighted, and in some cases T2* echo-planar imaging (EPI). Cases with a documented diagnosis of developmental venous anomaly in the original radiology reports were extracted. In addition, all studies showing susceptibility artifacts on sensitive sequences suggestive of hemorrhage, including intracranial hemorrhage, vascular malformations, or even questionable suspicion of hemosiderin, were systematically fully re-evaluated to identify developmental venous anomalies not categorized as such in the initial report. The methodology for this study followed the guidelines outlined in the STROBE statement checklist for observational studies [9].

### Imaging review

Fetal brain MRI studies were reviewed by a pediatric neuroradiologist with 25 years of experience and a PhD student with expertise in fetal neuroradiology (L.BS., S.L. respectively), each blinded to the other’s assessments. In cases of discrepancy, interpretations were reconciled through detailed consensus discussions with a third pediatric radiologist with 30 years of experience in fetal medicine (C.G.). They confirmed the diagnosis and evaluated the presence of developmental venous anomaly-related mechanical compression of adjacent structures, the number of developmental venous anomalies, their location, and the direction of drainage (superficial, deep, or mixed). Associated abnormalities on MRI were extracted as previously described, including draining venous varix thrombosis, adjacent parenchymal hemorrhage, gliosis, polymicrogyria, or restricted diffusion [1]. Fetuses with one or more such findings were classified as having complicated developmental venous anomalies, and the remainder as uncomplicated developmental venous anomalies [1]. In addition, fetuses with multiple developmental venous anomalies and those with parenchymal loss at the site of the developmental venous anomaly insertion were also considered to have a complicated developmental venous anomaly. A description of the imaging criteria to diagnose complicated developmental venous anomaly is presented in Supplementary Material 1. Other brain anomalies, when present on fetal MRI, were also documented, including malformations of cortical development (MCD) not directly related to the developmental venous anomaly location, corpus callosum and posterior fossa anomalies, ventriculomegaly, cysts, systemic abnormalities, and biometric data (including cerebral biparietal diameter, bone biparietal diameter, and trans-cerebellum diameter [10]).

Asymmetry of the medullary veins was defined as asymmetric hypointensity on susceptibility-sensitive sequences, reflecting prominence of medullary veins on one side compared with the contralateral hemisphere. This finding was recorded when the asymmetry was consistent across at least two orthogonal planes and not attributable to motion, partial volume effects, or adjacent parenchymal abnormalities.

### Magnetic resonance imaging acquisition

The acquisition of fetal brain MRI was performed using 3-T MRI scanners (Prisma, Vida, or Skyra; Siemens Healthineers, Erlangen, Germany) with a torso phased-array coil and a spine coil embedded in the scanner bed, while the participants were in a supine position. Following a localizing gradient-echo sequence, T2-weighted single-shot fast spin echo images (slice thickness: 2–4 mm) were obtained in the axial, coronal, and sagittal planes. T1-weighted imaging, diffusion-weighted imaging (DWI), and GRE-based T2-weighted sequences were acquired in at least one plane. In addition, in 50% of the fetuses, T2* echo-planar imaging (EPI) was acquired.

### Clinical and radiological data

Data regarding pregnancy history, sex, indication for fetal MRI, and gestational age at the time of fetal MRI were collected from the clinical records. Additional information on the presence of a parenchymal hyperechogenic focus, with or without a collecting vein, was obtained from prenatal and/or postnatal ultrasound (US) reports and from postnatal MRI when available.

### Statistical analysis

Demographics, imaging findings, and clinical characteristics were summarized as counts and percentages for categorical characteristics, mean and SD for normally distributed continuous variables, and median and interquartile range (IQR) for other distributions. Fisher’s exact test, *χ*^2^, Student’s *t*-test, and Mann–Whitney *U* test were used to compare angioarchitectural features and associated abnormalities in fetuses with complicated and uncomplicated developmental venous anomalies. Inter-rater agreement was assessed using Cohen’s *κ* coefficient, with 95% confidence intervals calculated from the asymptotic standard error. All statistical tests were two-tailed; a *P*<0.05 was considered statistically significant. Statistical analyses were conducted using SPSS v28 (IBM Corp., Armonk, NY).

## Results

**Fig. 1:**
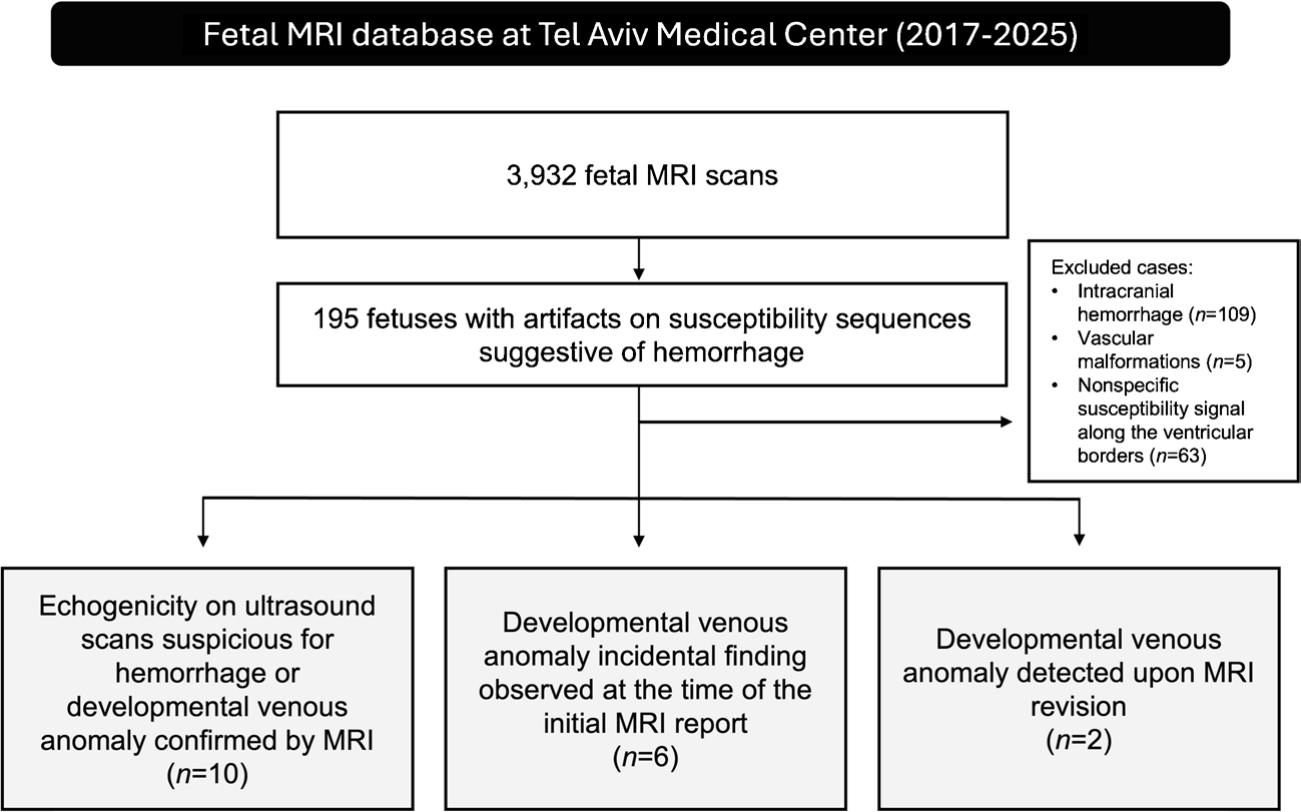
Flow diagram of case selection. Of 3,932 fetal MRI examinations performed between 2017 and 2025, 195 included cases with artifacts on susceptibility sequences suggestive of hemorrhage. Eighteen fetuses were diagnosed with developmental venous anomalies: ten referred due to echogenicity on ultrasound, suspicious for hemorrhage or developmental venous anomaly; six identified incidentally on initial MRI; and two detected upon retrospective review

Of 3,932 fetal MRIs, 195 (5%) fetuses were diagnosed with hemorrhage-sensitive sequences (GRE-based T2-weighted) in the initial report. Eighteen of them were identified with developmental venous anomalies, corresponding to an observed detection rate of 0.46% (18/3,932) at a tertiary hospital (Fig. 1). Inter-rater agreement was excellent (Cohen’s *κ*=0.83; 95% CI, 0.69–0.96; *P*<0.001), indicating near-perfect concordance between reviewers despite the relatively low prevalence of positive cases.

The median maternal age was 32 (IQR 27.5, 37) years, and the median gestational age at fetal MRI was 33 (IQR 32, 33) weeks. Of 17 fetuses with available sex data, 13 (76.5%) were males (Table 1). MRI sequence parameters and hemorrhage-sensitive techniques for each fetus are presented in Table 2.

**Table 1:** Demographic and magnetic resonance imaging characteristics of fetuses with uncomplicated and complicated developmental venous anomalies

	Total (*n*=18)	Uncomplicated developmental venous anomaly (*n*=10)	Complicated developmental venous anomaly (n=8)	*P-*value
Maternal age (years) at MRI median (IQR)	32 (27.75, 37)	34.5 (28.5, 37.25)	32 (27.5, 34.5)	0.46
Weeks of gestation at MRI median (IQR)	33 (32, 33)	33 (32, 33)	32.5 (30.5, 33)	0.41
Male gender (n=17)	13/17 (76.5)	8/10 (80)	5/7 (71.4)	1
**Supratentorial Biometry**^**a**^** n (%)**				0.79
>2 SD	5 (27.8)	2 (20)	3 (37.5)	
<2 SD	1 (5.6)	1 (10)	0	
**Developmental venous anomaly** side, right n (%)	14 (77.8)	7 (70)	7 (87.5)	0.59
**Location n (%)**				1
Frontal	10 (55.6)	5 (50)	5 (62.5)	
Parieto-Occipital	7 (38.8)	4 (40)	3 (37.5)	
Parietal	1 (5.6)	1 (10)	0	
**Drainage n (%)**				0.57
Deep	8 (44.4)	5 (50)	3 (37.5)	
Superficial	4 (22.2)	1 (10)	3 (37.5)	
Both	6 (33.3)	4 (40)	2 (25)	
Fisherman’s rope sign (%)	8 (44.4)	4 (40)	4 (50)	1
Hypointense T2 lining the ventricular wall (%)	12 (66.7)	8 (80)	4 (50)	0.20
**Asymmetric medullary veins n (%)**	7 (38.8)	5 (50)	2 (25)	0.37
Prominent side, right	3/7 (42.9)	3/5 (60)	0	0.43

*IQR* interquartile range, *SD* standard deviation

^a^Supratentorial biometry is based on the Bone Biparietal Diameter, Cerebral Biparietal Diameter and, Fronto Occipital Diameter using the norms according to Tilea et al. [10]

**Table 2:** Magnetic resonance imaging sequence parameters and hemorrhage-sensitive techniques used for evaluation of fetal developmental venous anomalies

Fetus no.	Developmental venous anomaly location	Developmental venous anomaly drainage	Year of MRI	GRE-based T2-weighted thickness (mm)	GRE-based T2-weighted TR/TE	GRE-based T2-weighted TR/TE resolution	T2* sequence (EPI) thickness (mm)	T2* sequence (EPI) TR/TE	T2* sequence (EPI) Resolution	Sagittal T2 thickness (mm)	Sagittal T2: TR/TE	Sagittal T2: resolution
1^a^	Frontal	Deep+superficial	2025	5.00	307/26	512*512	3.00	8,700/140	192*192	2.50	5.25/2.63	640*640
2^a^	Frontal	Superficial	2025	6.00	307/26	512*512	3.00	8,700/140	192*192	2.50	5.25/2.63	640*640
3	Frontal	Superficial	2024	5.50	307/26	512*512	3.00	8,700/140	192*192	2.50	5.25/2.63	640*640
4	Frontal	Deep+superficial	2024	3.00	1,000/44.35	176*176	ND	ND	ND	3.00	1,449/90	288*288
5	Frontal	Deep	2024	5.50	307/26	512*512	3.00	8,700/140	192*192	2.50	5.25/2.63	640*640
6	Frontal	Superficial	2024	5.50	307/26	512*512	3.00	8,700/140	192*192	2.50	5.25/2.63	640*640
7	Frontal	Deep+superficial	2023	5.00	307/26	512*512	3.00	8,700/140	192*192	3.00	5.25/2.63	640*640
8	Frontal	Superficial	2018	5.00	273/26	512*512	ND	ND	ND	2.50	4.94/2.47	512*512
9	Frontal	Deep	2017	1.00	307/26	512*512	ND	ND	ND	2.50	4.94/2.47	512*512
10^a^	Frontal	Deep	2017	5.00	344/26	512*512	ND	ND	ND	2.50	4.98/2.49	512*512
11	Parieto-occipital	Deep	2025	5.50	307/26	512*512	3.00	8,700/140	192*192	2.50	2,000/143	640*640
12	Parieto-occipital	Deep+superficial	2024	4.00	852/35	512*512	ND	ND	ND	4.00	110/96	256*256
13	Parieto-occipital	Deep+superficial	2024	5.00	375/26	512*512	ND	ND	ND	3.00	2,000/101	480*512
14^a^	Parieto-occipital	Deep	2024	5.00	307/26	512*512	ND	ND	ND	2.00	4.94/2.47	512*512
15	Parieto-occipital	Deep	2022	5.00	341/26	512*512	3.00	ND	ND	3.00	4.94/2.47	512*512
16	Parieto-occipital	Deep	2022	5.50	307/26	512*512	ND	8,700/140	192*192	3.00	4.94/2.47	512*512
17^a^	Parieto-occipital	Deep	2018	5.00	307/26	512*512	ND	ND	ND	2.50	4.98/2.49	512*512
18	Parietal	Deep+superficial	2025	5.00	307/26	512*512	3.00	8,700/140	192*192	2.50	5.25/2.63	640*640

*EPI* echo planar imaging, *GRE* gradient echo, *MRI* magnetic resonance imaging**,***ND* not done, *TE* echo time, *TR* repetition time

^a^Fetuses with unconfirmed developmental venous anomaly on prenatal or postnatal ultrasound

Of those identified with developmental venous anomaly on fetal MRI, ten (52.6%) were referred due to echogenicity on US scans, suspicious for hemorrhage or developmental venous anomaly. In the remaining eight (44.4%), referral was due to unrelated US findings (ventriculomegaly (5/8, 62.5%); periventricular pseudo-cysts; facial dysmorphism; and intrauterine growth restriction (1/8 12.5% each)). Of these, 6 (6/18, 33.3%) were incidental findings observed at the time of the initial report, and 2 (2/18, 11.1%) were recognized only upon revision (Fig. 1).

Prenatal and/or postnatal ultrasound confirmed stable parenchymal echogenicity, with or without a visible draining vein, in 13 of 16 fetuses (81.3%). Two developmental venous anomalies were excluded from this prevalence analysis because they were identified only on retrospective review; therefore, ultrasound confirmation was not part of the original diagnostic evaluation (#10, 17). Developmental venous anomalies were not confirmed on US in three cases: prenatal US was suboptimal in one fetus (#1), third trimester US (34 weeks and 36 weeks) in a fetus with frontal developmental venous anomaly with superficial drainage and associated parenchymal loss (#2) failed to detect the developmental venous anomaly and the parenchymal abnormality; ultrasound did not confirm the presence of a developmental venous anomaly in a frontal developmental venous anomaly with deep drainage in one case (#14), as the examination was unavailable for image review, and only the written report could be assessed. A detailed MRI across the entire cohort, demonstrating the spectrum of developmental venous anomalies radiological patterns, is presented in the Supplementary Material 2.

### Fetal developmental venous anomaly patterns and location

**Fig. 2:**
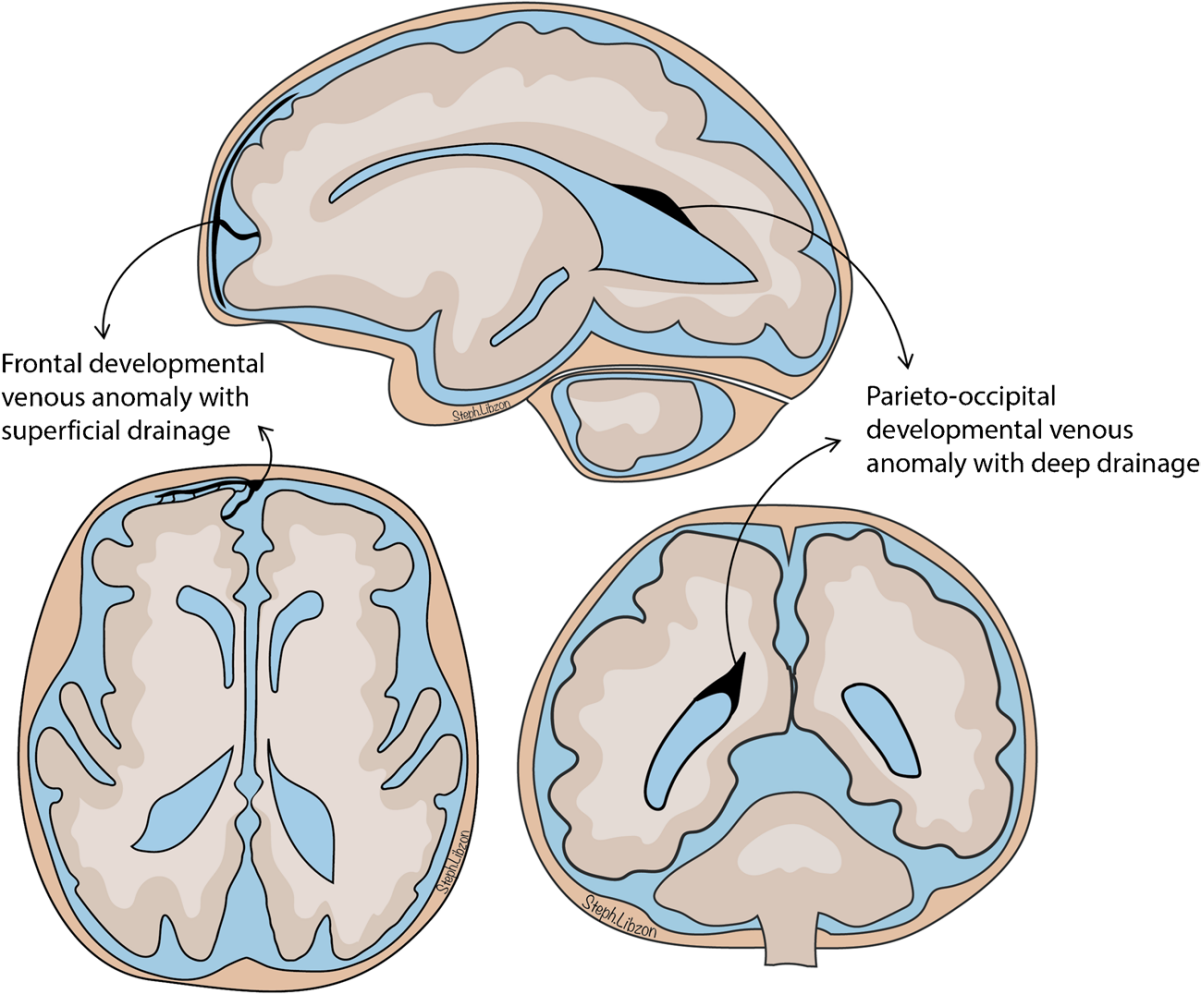
Illustration of the two fetal developmental venous anomaly patterns shown in three planes: (**a**) parasagittal, (**b**) axial, and (**c**) coronal: frontal developmental venous anomaly with superficial drainage, and parenchymal loss in the insertion of the developmental venous anomaly, and parieto-occipital developmental venous anomaly with deep drainage seen as irregular thickening of the ventricular wall. **c** In the coronal plane, developmental venous anomaly with deep drainage in the parieto-occipital region appears as lesions “enveloping” the superior margin of the lateral ventricle

Developmental venous anomalies were most readily identified on sequences sensitive to hemorrhage, appearing as a parenchymal blooming artifact, in the region of the collecting veins. The classical “Caput Medusa” configuration was not observed. On T2-weighted imaging, at least one of the following configurations was observed: (1) small linear hypointense lesions in the surrounding white matter; (2) hypointense linear structures that “hug” the superior margin of the lateral ventricle at the atrial level; and (3) visualization of a superficial draining vein within the extra-axial spaces, sometimes seen crossing the adjacent parenchyma. These findings were primarily identified when high-resolution, thin slices were available, and usually without clear corresponding changes on T1-weighted imaging (Fig. 2).

Developmental venous anomalies were most frequently frontal (10/18, 55.6%), followed by parieto-occipital (7/18, 38.9%) and parietal (1/18, 5.6%). Most demonstrated deep drainage (8/18, 44.4%), followed by superficial (4/18, 22.2%), and 6 (33.3%) exhibited both draining patterns. Among eight cases diagnosed by MRI, five developmental venous anomalies were in the parieto-occipital lobe, showing deep (*n*=3) or combined (*n*=2) drainage, and three were in the frontal lobe with superficial (*n*=1), deep (*n*=1), and combined (*n*=1) drainage. Most were observed on the right side (14/18, 77.8%). Deep drainage involvement was observed in all cases located in the parieto-occipital region. Figure 2 presents an illustration of the two main locations of developmental venous anomaly found in fetuses. MRI characteristics of developmental venous anomalies are presented in Table 1.

**Fig. 3:**
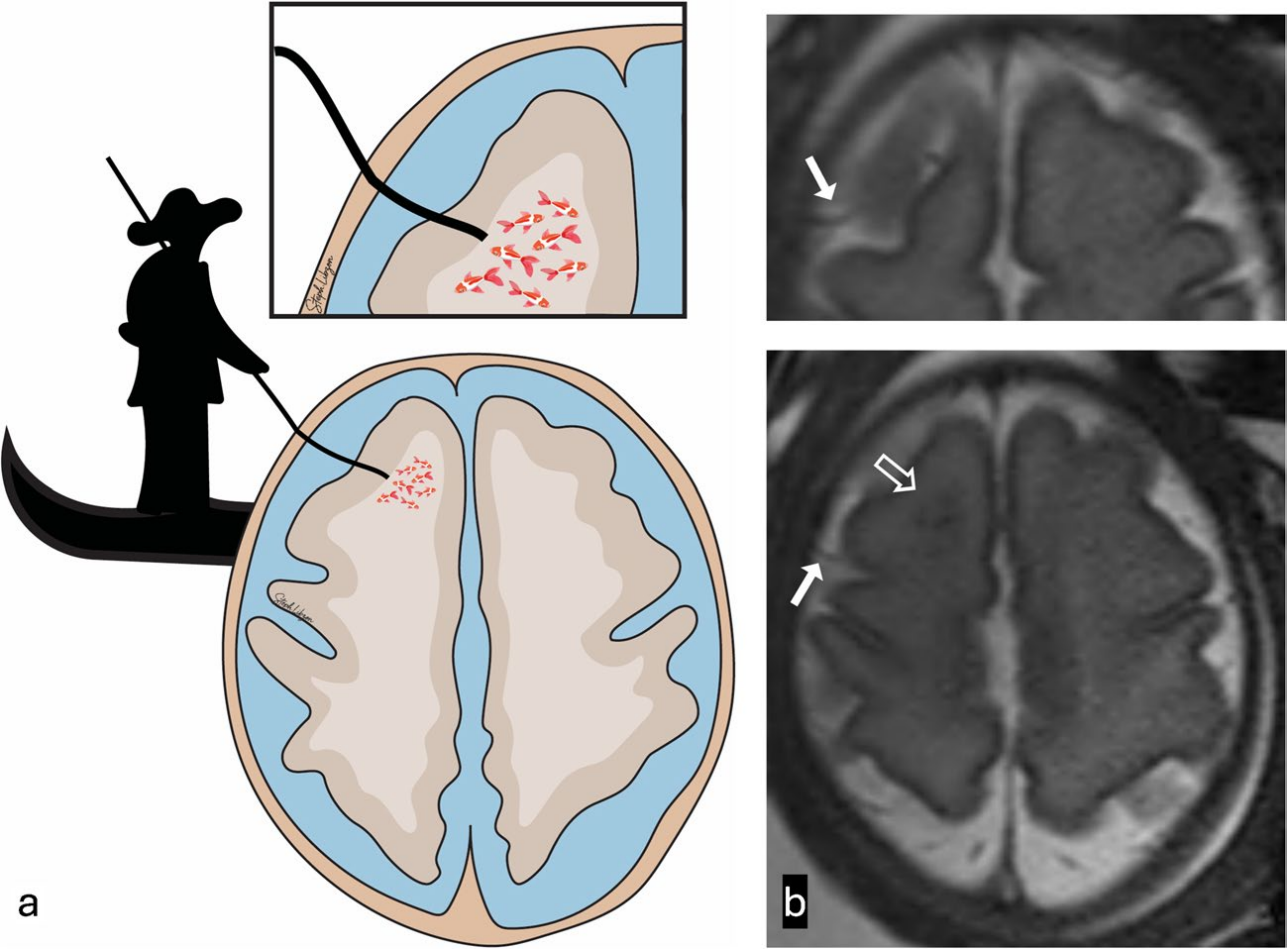
**a** Illustration of the “fisherman’s rope” sign in an axial plane: the rope-like superficial vein enters the right frontal parenchyma. At the same time, Japanese fish symbolize small T2 parenchymal hypointensities, barely identified. Magnetic resonance images in a male fetus (#3) at 29+6 weeks’ gestational age with a frontal developmental venous anomaly and superficial drainage. **b** Axial T2-weighted images show the superficial vein (*arrow*) and small T2 hypointensities (*empty arrow*) in the right frontal lobe

Superficial drainage, typically in the frontal lobe, when parasagittal thin T2-weighted imaging (2.5 mm) was acquired, the collecting vein transversing the adjacent extra-axial fluid could be depicted. This pattern of a collecting vein with adjacent small linear hypointense T2, parenchymal lesions resembles a Japanese fisherman’s rope (“fisherman’s rope” sign) (Fig. 3).

**Fig. 4:**
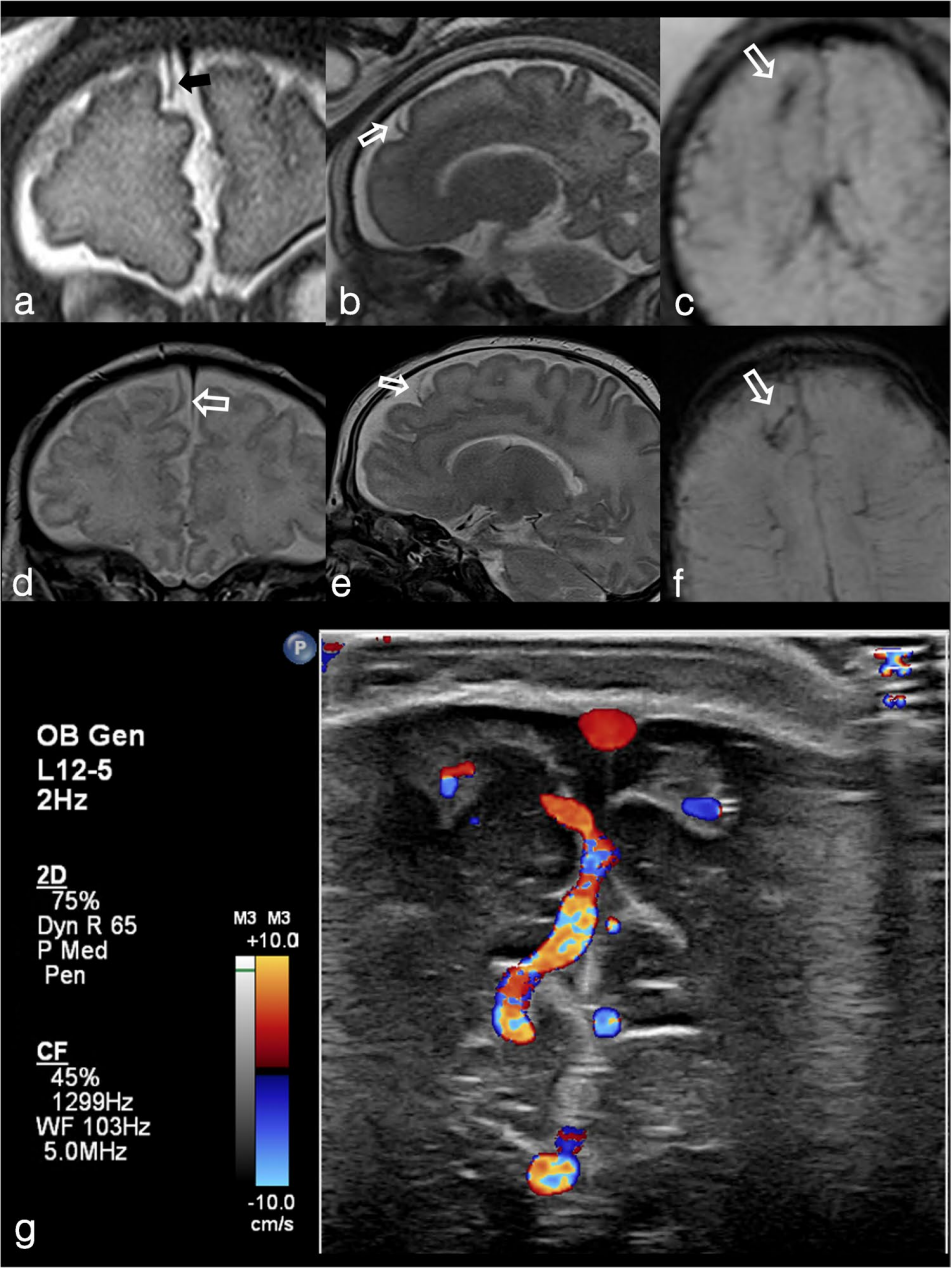
Magnetic resonance images of a developmental venous anomaly with superficial drainage. Magnetic resonance images in a male fetus (#6) at 33 weeks’ gestational age (**a**-**c**), postnatally at 24 days of life (**d**-**f**), and postnatal Doppler ultrasound at 54 days of life (**g**) demonstrate complicated developmental venous anomaly in the frontal lobe, with superficial drainage and parenchymal loss in the insertion of the draining vein. **a**,** d** Coronal and (**b**,** e**) parasagittal T2-weighted thin slices show the superficial vein transversing the right frontal parenchyma (*arrow*) with parenchymal loss in the insertion of the developmental venous anomaly. **c**, **f** Transverse susceptibility-weighted image shows blooming artifacts corresponding to the developmental venous anomaly (*empty arrow*). The classic “Caput Medusa” is shown only on the postnatal susceptibility-weighted imaging MRI (**f**). **g** Coronal postnatal Doppler ultrasound at 54 days of life demonstrates the insertion of the draining vein in the right frontal lobe

The “fisherman’s rope” sign was more frequently observed in fetuses with enlarged extra-axial spaces (5/6 83.3% vs 3/12 25%, *P*=0.04) (Fig. 4).

**Fig. 5:**
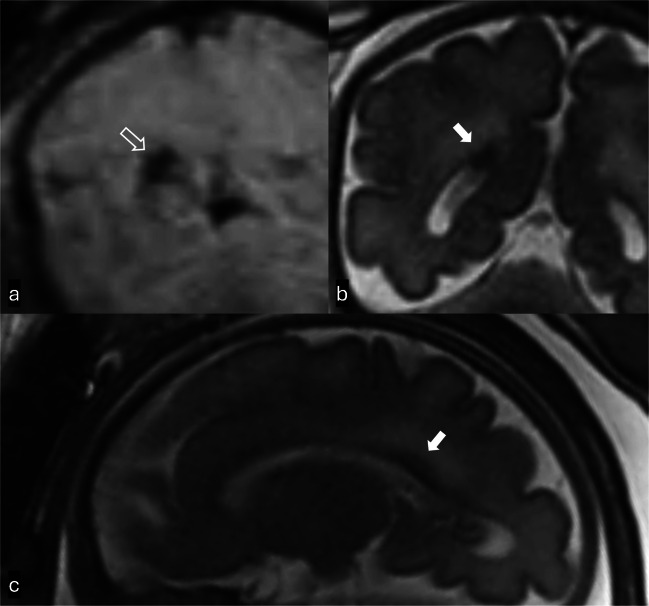
Magnetic resonance images of a developmental venous anomaly with deep drainage. Magnetic resonance images in a female fetus (#15) at 33 weeks’ gestational age demonstrate parieto-occipital developmental venous anomaly and deep drainage. **a** Coronal susceptibility-weighted image shows blooming artifacts corresponding to the developmental venous anomaly (*empty arrow*). **b** Coronal T2-weighted images showing hypointense lesions “enveloping” the superior margin of the right lateral ventricle at the level of the atrium (*arrow*). **c** Parasagittal image demonstrating small hypointense lesions lining the ventricular wall (*arrow*)

Developmental venous anomalies with deep drainage (*n*=14) demonstrated small, focal, hypointense T2 irregular thickening of the ventricular wall. On coronal images, these lesions presented as hypointense areas that “hug” or envelop the superior margin of the lateral ventricle at the atrium level, without associated parenchymal loss or irregularities of the ventricular wall (Fig. 5).

In our cohort, developmental venous anomalies demonstrating solely superficial drainage were exclusively in the frontal lobe (4/10 (40%) vs 0, *P*=0.09). These cases were more often associated with focal parenchymal loss at the developmental venous anomaly insertion site (3/4 (75%) vs 3/14 (21.4%), *P*=0.08) (Fig. 4).

Eight fetuses (44.4%) were diagnosed with complicated developmental venous anomalies, including parenchymal loss in the insertion of the developmental venous anomaly in 33.3% (6/18), multiple developmental venous anomalies in 16.7% (3/18), draining venous varix thrombosis (2/18, 11.1%), and adjacent parenchymal hemorrhage (2/18, 11.1%). Thrombosis of a draining venous varix was associated with multiple developmental venous anomalies (2/3, 66.7% vs 0/15, *P*=0.02).

Five fetuses (27.8%) had concurrent ventriculomegaly (mild in 2 cases, moderate in 2 cases, and severe in 1 case): in 3 fetuses, the developmental venous anomaly was contralateral to the enlarged ventricle, in one, it was ipsilateral, and in one, the ventriculomegaly was bilateral and severe. Seven fetuses (7/18, 38.9%) had asymmetry of the medullary veins, most of them (6/7, 85.7%) observed on the contralateral side of the developmental venous anomaly. Additional brain anomalies, not directly related to the developmental venous anomaly location, included abnormal biometry in 6 fetuses: 5 (27.8%) had large head biometry (>2 SD above the mean), and 1 (5.6%) had small biometry (<2 SD below the mean). Enlarged extra-axial fluid spaces were present in 6 (33.3%) fetuses, cortical asymmetry, and white matter changes in one case each (5.6%).

All fetuses (100%) were liveborn. Four underwent more than one fetal MRI during pregnancy, which confirmed stability of the developmental venous anomaly over time. Seven neonates underwent postnatal US, and five of them underwent postnatal brain MRI at a median age of 12 days (IQR 8.5, 20). All postnatal MRI were obtained without contrast except for one case (#4), confirming the stable appearance of prenatal findings in all (5/5) (Fig. 4). All of them (5/5) demonstrated the classical appearance of “Caput Medusa” on SWI that was not recognized in the fetal MRI (Fig. 4). In all but one case, the developmental venous anomalies were stable in configuration and number. One neonate (#4), who had a complicated developmental venous anomaly prenatally (two developmental venous anomalies and varix thrombosis), was found to have multiple developmental venous anomalies on postnatal MRI, without parenchymal hemorrhage, and is currently under investigation.

## Discussion

This study reports the prevalence of fetal developmental venous anomalies, based on the largest systematic MRI description to date, demonstrating that they can be consistently recognized in utero with distinct and reproducible imaging features. The observed detection rate in our large fetal cohort, from a tertiary hospital, was 0.46%. In nearly half of the cases (8/18, 44.4%), the diagnosis of developmental venous anomaly was made exclusively by fetal MRI, highlighting the unique diagnostic advantage of fetal MRI for venous anomalies. In addition, two characteristic MRI patterns were identified: the “fisherman’s-rope” sign in superficial developmental venous anomalies and the ventricular-wall T2 hypointensity in deep developmental venous anomalies. These patterns can help neuroradiologists differentiate developmental venous anomalies from hemorrhage and other vascular malformations, thereby improving the accuracy of prenatal identification and classification.

### Prevalence of fetal developmental venous anomalies

The observed prevalence in our fetal cohort of 0.46% is lower than that reported in neonates (0.8–1.9%) and substantially lower than the prevalence observed in older children and adults (5–10%) [4, 11–14]. This difference likely reflects technical limitations of fetal MRI, motion artifacts, limited spatial resolution, and absence of intravenous contrast, as well as the possibility that the prevalence of developmental venous anomalies increases with age [13].

### Location and laterality

As in prior neonatal reports, frontal developmental venous anomalies were most common [1, 11, 12], followed by parieto-occipital and, rarely, parietal locations. Right-sided predominance (78%) was also consistent with postnatal studies [1, 12], although the biological significance remains unclear. Several developmental venous anomalies were detected incidentally, highlighting the importance of scrutinizing venous structures even when MRI is performed for unrelated indications.

The absence of infratentorial developmental venous anomalies likely reflects both their rarity [15] and the technical challenges in detecting susceptibility-related changes in the posterior fossa on fetal MRI. One excluded case demonstrated cerebellar susceptibility artifacts initially interpreted as capillary telangiectasia, later raising suspicion for an associated or atypically draining developmental venous anomaly, with arteriovenous malformation considered in the differential diagnosis. This case underscores the ongoing diagnostic challenge in distinguishing venous anomalies from other vascular malformations [6, 16].

### Characteristic magnetic resonance imaging patterns

**Fig. 6:**
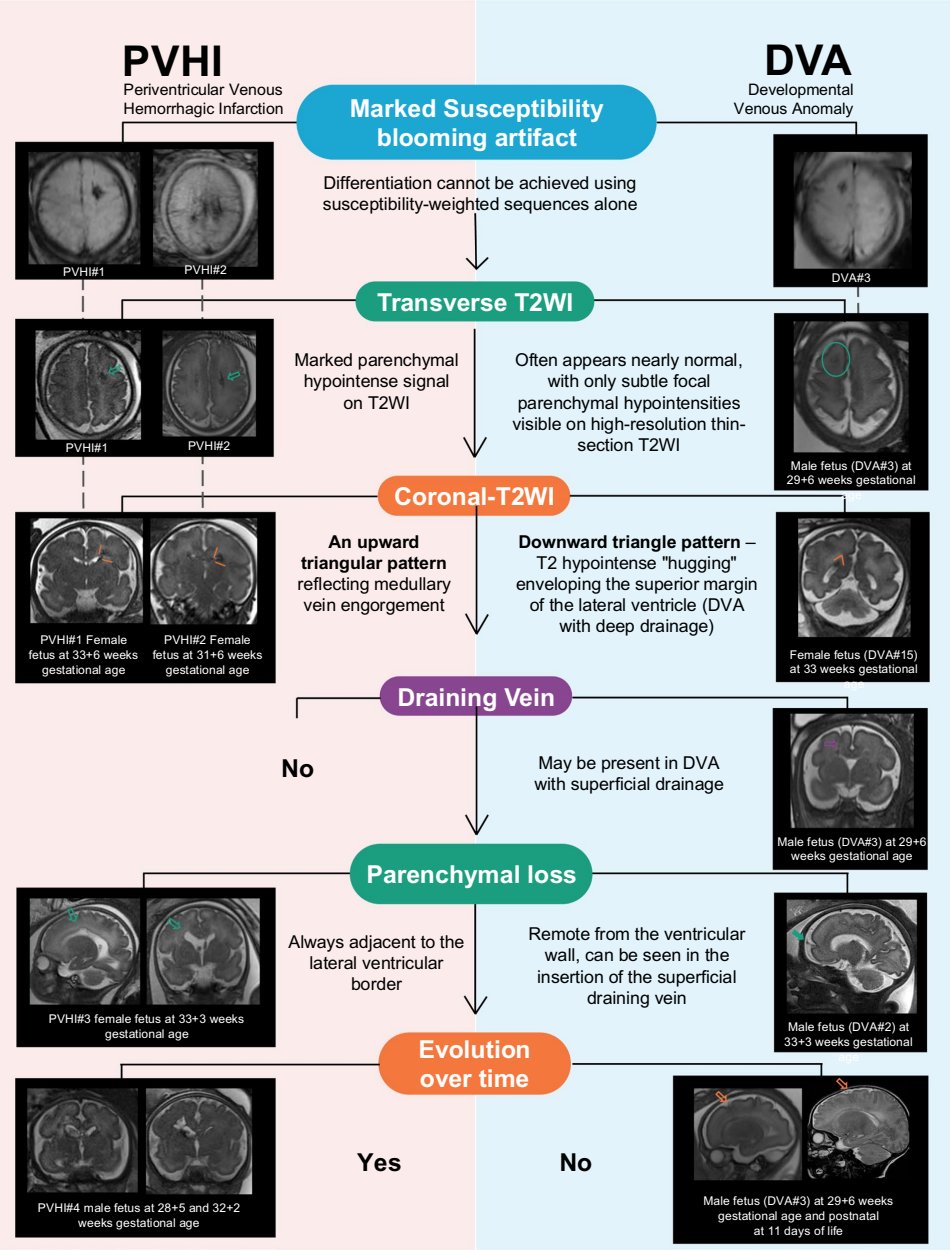
Diagnostic flowchart for differentiating developmental venous anomalies from periventricular venous hemorrhagic infarction on fetal magnetic resonance imaging. The diagram emphasizes the limitations of susceptibility-weighted sequences alone and the added diagnostic value of high-resolution T2-weighted and coronal T2-weighted imaging in evaluating venous architecture, parenchymal involvement, and lesion evolution

Blooming artifacts along the collecting vein were best visualized on hemorrhage-sensitive sequences, while linear T2 hypointensities were occasionally identified in the surrounding white matter. Frontal developmental venous anomalies with superficial drainage often exhibited the “fisherman’s rope sign,” particularly when adjacent extra-axial fluid spaces were enlarged. Deep-drainage developmental venous anomalies demonstrated T2 hypointense thickening appeared as irregular lesions anchored to the ventricular wall, often enveloping the atrium, and emerged as a reproducible pattern. This distinctive pattern has not been previously described in developmental venous anomalies and needs to be differentiated from hemorrhage, as the prognostic and counseling implications differ substantially (Fig. 6). The discrepancy between the hyperechogenic lesion on ultrasound and the prominent hypointensity on hemorrhage-sensitive sequences on MRI, in contrast to the delicate T2 hypointensity on fetal MRI, both of which demonstrate no evolution over time, provides a key diagnostic indicator supporting the diagnosis of a developmental venous anomaly.

Contrary to prior assumptions that developmental venous anomalies cannot be visualized on T2-weighted MRI sequences [6], our data show that high-resolution, thin-slice imaging consistently depicts the superficial draining vein in the extra-axial spaces and sometimes in the adjacent parenchyma (Figs. 3 and 4). In addition, on T2-weighted imaging, small linear hypointense lesions in the surrounding white matter were occasionally visible, primarily when high-resolution, thin slices were available (Fig. 3). The discrepancy between a prominent blooming artifact and delicate parenchymal T2 hypointensities serves as an additional diagnostic indicator. Although contrast-enhanced MRI remains the postnatal gold standard for developmental venous anomaly identification, our findings confirm that hemorrhage-sensitive sequences GRE-based T2-weighted and T2*-weighted EPI sequences provide sufficient diagnostic information in the prenatal setting [17]. This supports the routine inclusion of these sequences in fetal MRI protocols. While none of the fetuses demonstrated the classic “caput medusae” appearance on fetal MRI, this finding was present in all neonates who underwent postnatal MRI (*n*=5) (Fig. 4). This discrepancy may be related to the limited sensitivity of fetal hemorrhage-sensitive GRE-based T2-weighted sequences compared with the higher spatial resolution and greater susceptibility sensitivity of postnatal SWI sequences.

### Developmental venous anomalies versus periventricular venous hemorrhagic infarction

Distinguishing developmental venous anomalies from periventricular venous hemorrhagic infarction (PVHI) is critical for accurate diagnosis and counseling, given their distinct pathophysiology, prognosis, and clinical implications. Although both entities may demonstrate susceptibility-related hypointensity adjacent to the ventricular wall, particularly on GRE-based T2-weighted, and T2* EPI sequences, susceptibility findings alone are insufficient for differentiation. Careful assessment of angioarchitectural features and associated parenchymal and ventricular changes is therefore essential.

On thin-section, high-resolution T2-weighted sequences, PVHI typically demonstrates marked parenchymal hypointensity reflecting hemorrhagic products, whereas in developmental venous anomalies, T2-weighted sequences often appear nearly normal, with only subtle and sometimes barely perceptible focal T2 hypointensities (“the fisherman-rope sign”). Coronal T2 imaging further aids differentiation: PVHI is characterized by medullary vein engorgement forming an upward triangular configuration extending toward the ventricular margin, while deep developmental venous anomalies typically display a downward triangular pattern, with hypointense linear structures that envelop or “hug” the superior margin of the lateral ventricle at the atrial level.

When present, parenchymal injury also differs among entities. In PVHI, parenchymal loss is consistently adjacent to the lateral ventricular border, reflecting venous infarction, whereas in developmental venous anomalies, parenchymal changes, when present, are often remote from the ventricle and usually located at the insertion site of a superficial draining vein. Another important factor is the MRI abnormality evolution over time; developmental venous anomalies tend to be stable, whereas the signal intensity of PVHI evolves according to the age of hemorrhage and is often accompanied by evolving focal lateral ventricle abnormality, in some severe cases, may evolve into a porencephalic cyst. These findings are supported by the stable parenchymal echogenicity demonstrated on US in cases with developmental venous anomalies.

These distinctions highlight the importance of combining susceptibility-weighted imaging with thin-section, high-resolution T2-weighted sequences and follow-up imaging to reliably establish the diagnosis of developmental venous anomaly versus PVHI in the prenatal setting (Fig. 6).

### Complicated developmental venous anomalies

Nearly half of the fetuses had a complicated developmental venous anomaly. Although causality cannot be established, these associations suggest that developmental venous anomalies may coexist with parenchymal vulnerability or altered venous drainage. Frontal developmental venous anomalies with superficial drainage were often linked with focal parenchymal loss at the venous insertion site (Fig. 4). While not statistically significant, this consistent association suggests a topographic or hemodynamic relationship. Thrombosis of a draining venous varix was linked with multiple developmental venous anomalies, identifying a subgroup at potential risk for vascular complications. Recognition of this pattern may warrant closer prenatal and postnatal surveillance. In our cohort, no developmental venous anomalies were associated with cavernous malformations, as has been previously described in both pediatric [1, 11, 18] and adult [19, 20] populations.

### Additional brain magnetic resonance imaging findings

Five fetuses demonstrated ventriculomegaly, contralateral to the developmental venous anomaly in most cases (3/5, 60%). Ipsilateral and bilateral ventriculomegaly were seen in only one case each. This contrasts with prior reports [5] describing an ipsilateral developmental venous anomaly-ventriculomegaly association with parenchymal loss. Although not observed in our cohort, obstructive hydrocephalus secondary to aqueductal stenosis caused by a developmental venous anomaly has been previously reported, including in cases diagnosed prenatally with ventriculomegaly [21]. Contralateral medullary vein asymmetry was observed in 85.7% of cases (6/7) (Supplementary Material 3). This may reflect venous flow redistribution in which impaired outflow through the developmental venous anomaly is compensated by contralateral venous dilatation, potentially increasing venous pressures and contributing to ventricular enlargement. While speculative in the fetal setting, this mechanism is supported by postnatal hemodynamic studies of symptomatic developmental venous anomalies [22]. Although this study was not designed to assess the clinical impact of fetal developmental venous anomalies, our findings suggest that these anomalies should be carefully evaluated for associated abnormalities and secondary effects, such as parenchymal loss.

This study, conducted in a single tertiary hospital, is limited by its retrospective design and small sample size, and by the inclusion of fetuses referred for abnormal brain US may introduce selection bias. In addition, the absence of contrast-enhanced sequences could have led to the under-recognition of subtle developmental venous anomalies. Not all cases had confirmatory imaging, reflecting the retrospective nature of the study and technical limitations, including incomplete imaging datasets and the inherent challenges in visualizing small vascular structures with ultrasound. These factors may limit definitive validation in a small subset of cases. Further studies in larger fetal cohorts with a wider range of gestational ages are needed to assess the reproducibility, specificity, and diagnostic accuracy of the findings in diagnosing fetal developmental venous anomalies.

## Conclusions

This retrospective study provides the prevalence of fetal developmental venous anomaly in a large fetal cohort and highlights the indispensable role of MRI in their detection. We identified two distinct reproducible MRI patterns, with particular emphasis on hemorrhage-sensitive sequences - GRE-based T2-weighted and T2*EPI, combined with high-resolution, thin slices, T2-weighted imaging, and imaging follow-up, which improve diagnostic confidence and support a more standardized approach to fetal MRI assessment.

## Supplementary Information

Below is the link to the electronic supplementary material.

Supplementary file1 (PDF 14.5 KB)

Supplementary file2 (PDF 687 KB)

Supplementary file3 (PDF 118 KB)

## Data Availability

Data generated or analyzed during the study are available from the corresponding author upon request.

## Abbreviations

IQR: Interquartile range
GRE: Gradient echo
EPI: Echo-planar imaging
SWI: Susceptibility-weighted imaging

## References

[1] Geraldo AF, Messina SS, Tortora D, et al (2020) Neonatal developmental venous anomalies: clinicoradiologic characterization and follow-up. AJNR Am J Neuroradiol 41:2370–2376 33093132 10.3174/ajnr.A6829 PMC7963246

[2] Linscott LL, Leach JL, Jones BV, Abruzzo TA (2016) Developmental venous anomalies of the brain in children — imaging spectrum and update. Pediatr Radiol 46:394–406; quiz 391–393 10.1007/s00247-015-3525-3 26795616

[3] Lee C, Pennington MA, Kenney CM 3rd (1996) MR evaluation of developmental venous anomalies: medullary venous anatomy of venous angiomas. AJNR Am J Neuroradiol 17:61–70 8770251 PMC8337971

[4] Kıyak V, Beyhan M, Gökçe E (2025) Magnetic resonance imaging findings of cerebral venous malformations. Neurol Sci 46:1721–1732 39641890 10.1007/s10072-024-07912-y

[5] Geraldo AF, Melo M, Monteiro D, et al (2018) Developmental venous anomaly depicted incidentally in fetal MRI and confirmed in post-natal MRI. Neuroradiology 60:993–994 30155642 10.1007/s00234-018-2089-y

[6] Krajden Haratz K, Peled A, Weizman B, et al (2018) Unique imaging features enabling the prenatal diagnosis of developmental venous anomalies: a persistent echogenic brain lesion drained by a collecting vein in contrast with normal brain parenchyma on MRI. Fetal Diagn Ther 43:53–60 28624828 10.1159/000464247

[7] Brinjikji W, Lanzino G (2019) Letter regarding “Developmental venous anomaly depicted incidentally in fetal MRI and confirmed in post-natal MRI.” Neuroradiology 61:9 30382293 10.1007/s00234-018-2123-0

[8] Ruíz DS, Yilmaz H, Gailloud P (2009) Cerebral developmental venous anomalies: current concepts. Ann Neurol 66:271–283 19798638 10.1002/ana.21754

[9] Cuschieri S (2019) The STROBE guidelines. Saudi J Anaesth 13(Suppl 1):S31–S34 10.4103/sja.SJA_543_18 PMC6398292 30930717

[10] Tilea B, Alberti C, Adamsbaum C, et al (2009) Cerebral biometry in fetal magnetic resonance imaging: new reference data. Ultrasound Obstet Gynecol 33:173–181 19172662 10.1002/uog.6276

[11] Silva AHD, Wijesinghe H, Lo WB, et al (2020) Paediatric developmental venous anomalies (DVAs): how often do they bleed and where? Childs Nerv Syst 36:1435–1443 31900628 10.1007/s00381-019-04460-1

[12] Gökçe E, Acu B, Beyhan M, et al (2014) Magnetic resonance imaging findings of developmental venous anomalies. Clin Neuroradiol 24:135–143 24240482 10.1007/s00062-013-0235-9

[13] Brinjikji W, El-Rida El-Masri A, Wald JT, Lanzino G (2017) Prevalence of developmental venous anomalies increases with age. Stroke 48:1997–1999 28536179 10.1161/STROKEAHA.116.016145

[14] Carney O, Hughes E, Tusor N, et al (2021) Incidental findings on brain MR imaging of asymptomatic term neonates in the Developing Human Connectome Project. EClinicalMedicine 38:100984 34355154 10.1016/j.eclinm.2021.100984 PMC8322308

[15] Horsch S, Govaert P, Cowan FM, et al (2014) Developmental venous anomaly in the newborn brain. Neuroradiology 56:579–588 24756165 10.1007/s00234-014-1367-6

[16] Guibaud L, Garel C, Annie B, et al (2003) Prenatal diagnosis of capillary telangiectasia of the cerebellum—ultrasound and MRI features. Prenat Diagn 23:791–796 14558021 10.1002/pd.695

[17] Rauschenbach L, Santos AN, Rodemerk J, et al (2025) Detection of cerebral cavernous malformation associated developmental venous anomalies in gradient-echo and susceptibility-weighted magnetic resonance imaging: can we skip the contrast? Neuroradiology. 10.1007/s00234-025-03666-2 40512378 10.1007/s00234-025-03666-2 PMC12494666

[18] Linscott LL, Leach JL, Zhang B, Jones BV (2014) Brain parenchymal signal abnormalities associated with developmental venous anomalies in children and young adults. AJNR Am J Neuroradiol 35:1600–1607 24831595 10.3174/ajnr.A3960 PMC7964448

[19] San Millán Ruíz D, Delavelle J, Yilmaz H, et al (2007) Parenchymal abnormalities associated with developmental venous anomalies. Neuroradiology 49:987–995 17703296 10.1007/s00234-007-0279-0

[20] Zhang S, Ma L, Wu C, et al (2020) A rupture risk analysis of cerebral cavernous malformation associated with developmental venous anomaly using susceptibility-weighted imaging. Neuroradiology 62:39–47 31482190 10.1007/s00234-019-02274-1

[21] Mertiri L, Singh V, Gentile F, et al (2025) Obstructive hydrocephalus due to developmental venous anomalies: a pediatric imaging case series. Neuroradiology 67:493–497 39466406 10.1007/s00234-024-03484-y

[22] Pereira VM, Geibprasert S, Krings T, et al (2008) Pathomechanisms of symptomatic developmental venous anomalies. Stroke 39:3201–3215 18988912 10.1161/STROKEAHA.108.521799

