# Supplementary material for: Developmental venous anomalies on fetal magnetic resonance imaging: prevalence and reproducible radiological phenotypes
Source: Pediatr Radiol. 2026 Jul 13;56(8):1776–88. doi: 10.1007/s00247-026-06704-0 (PMC13407610; doi:10.1007/s00247-026-06704-0)
Supplement: Supplementary file 1 — Supplementary file1 (PDF 14.5 KB) [file 247_2026_6704_MOESM1_ESM.pdf]

Supplementary Material 1

Description of the imaging criteria to diagnose complicated developmental venous anomalies

Complications of developmental venous anomalies were defined as follows:

1) Parenchymal loss was defined as focal volume loss or cavitation in the drainage territory of the developmental venous anomaly

2) Parenchymal hemorrhage was identified by a focal susceptibility signal on GRE-based T2WI and/or T2*-weighted sequences, with corresponding parenchymal signal abnormality in other sequences (T1 and/or T2-weighted imaging ) evolving over time.

3) Venous thrombosis was defined as an abnormal signal, usually with focal dilatation (varix) of the draining vein.

4) Multiple developmental venous anomalies were defined as the presence of more than one distinct venous anomaly.
