# Supplementary material for: Developmental venous anomalies on fetal magnetic resonance imaging: prevalence and reproducible radiological phenotypes
Source: Pediatr Radiol. 2026 Jul 13;56(8):1776–88. doi: 10.1007/s00247-026-06704-0 (PMC13407610; doi:10.1007/s00247-026-06704-0)
Supplement: Supplementary file 2 — Supplementary file2 (PDF 687 KB) [file 247_2026_6704_MOESM2_ESM.pdf]

Supplementary Material 2: Fetal magnetic resonance images spectrum of developmental venous anomalies in the entire cohort

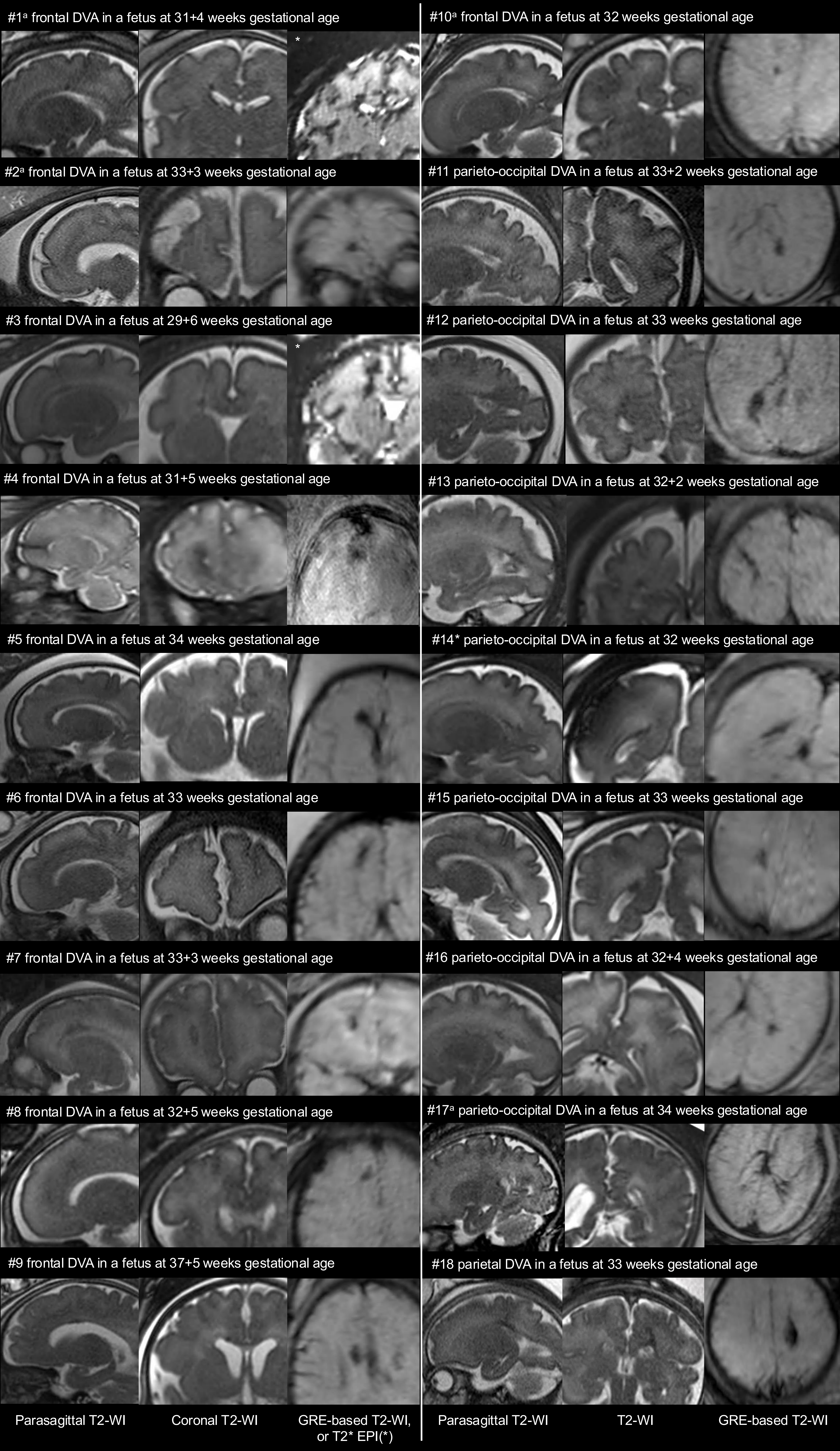

Supplementary Material 2:

Fetal magnetic resonance images spectrum of developmental venous anomalies in the entire cohort

Detailed magnetic resonance imaging (MRI) across the entire cohort, demonstrating the spectrum of developmental venous anomalies radiological patterns. Each fetus is shown with three MRI sequences, including (from right to left) midsagittal T2-weighted imaging (T2WI), coronal T2WI, and axial or coronal gradient-echo (GRE)-based T2WI. In two fetuses (#1 and #3), T2*-weighted echo-planar imaging (EPI) is presented instead (*asterisk*). [a] Fetuses with unconfirmed developmental venous anomalies on pre- or post-natal ultrasound
