# Supplementary material for: Developmental venous anomalies on fetal magnetic resonance imaging: prevalence and reproducible radiological phenotypes
Source: Pediatr Radiol. 2026 Jul 13;56(8):1776–88. doi: 10.1007/s00247-026-06704-0 (PMC13407610; doi:10.1007/s00247-026-06704-0)
Supplement: Supplementary file 3 — Supplementary file3 (PDF 118 KB) [file 247_2026_6704_MOESM3_ESM.pdf]

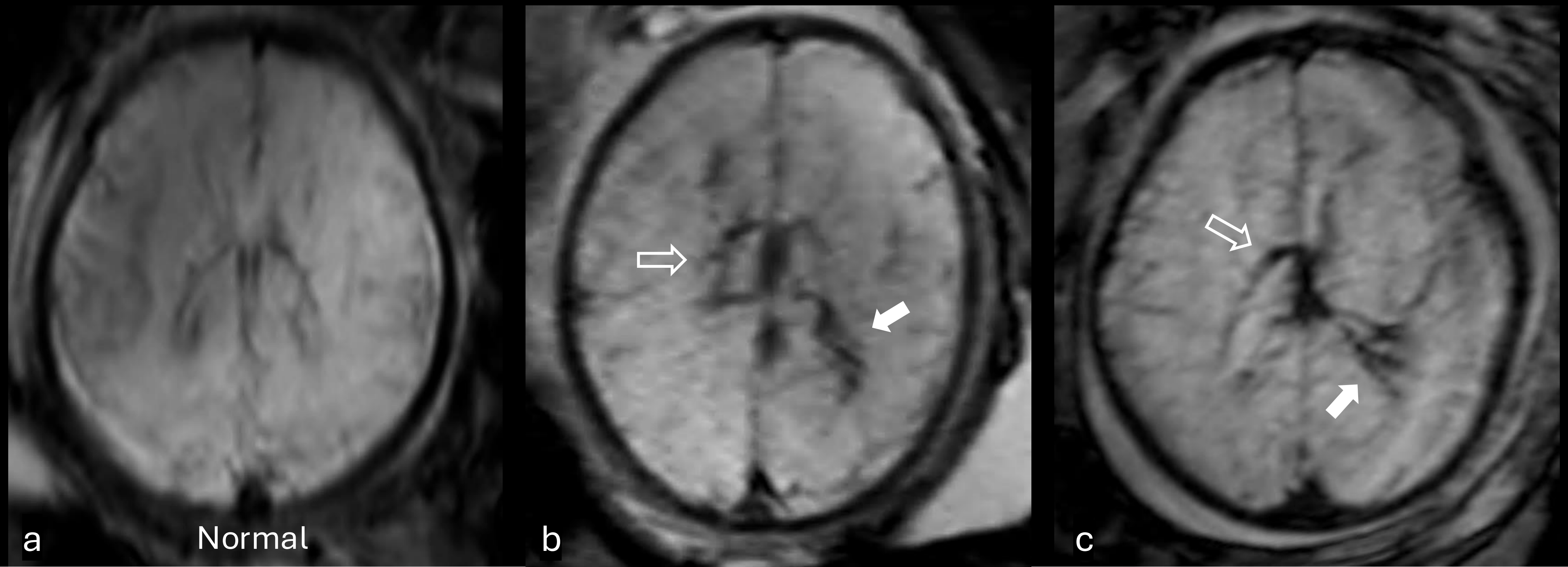
a
Normal
b
c

Supplementary Material 3

Magnetic resonance images demonstrating the medullary veins on transverse susceptibility-weighted images.

a reference image of the symmetric appearance of the veins in a normal male fetus at 32 weeks of gestational age. (**b-c**) Asymmetric medullary veins on the right (*empty arrow*), contralateral to the parieto-occipital developmental venous anomaly on the left (arrow) in two fetuses: **b** male fetus at 34 weeks of gestational age, and **c** fetus at 34 weeks of gestational age.
